# 

**Published:** 1908-12-12

**Authors:** 


THE
### GRADUATES' MEDICAL SCHOOLS.
DIARY FOR WEEK DEC. 14 TO DEC. 18.

**THE HOSPITAL FOR SICK CHILDREN, Great Ormond Street, W.C.**
**At 4 p.m.**
**Dec. 17,** Mr. Collum, **The Administration of Anæsthetics to Children.**

**NORTH-EAST LONDON POST-GRADUATE COLLEGE, Prince of Wales's General Hospital, Tottenham, N.**
**At 4.30 p.m.**
**Dec. 15,** Dr. Murray Leslie, **Recent Discoveries in Tuberculosis.**
**Dec. 17,** Dr. A. G. Auld, **Methods of Diagnosis applicable to the Abdominal Organs.**

**NATIONAL HOSPITAL FOR THE PARALYSED AND EPILEPTIC, Queen Sq., Bloomsbury, W.C.**
**At 3.30 p.m.**
**Dec. 15,** Dr. Grainger Stewart, **Spinal Caries.**

**THE POST-GRADUATE COLLEGE, West London Hospital, Hammersmith, W.**
**At 10 a.m.**
**Dec. 14 and 17,** Mr. Etherington Smith, **Surgical Demonstration.**
**At 12 noon.**
**Dec. 14,** Dr. Low, **Pathological Demonstration.**
**At 12.15 p.m.**
**Dec. 16 and 18,** Dr. Pritchard, **Practical Medicine.**
**At 5 p.m.**
**Dec. 14,** Mr. Dunn, **Eye Diseases.**
**Dec. 15,** Mr. Bidwell, **The Causes of Disappointing Results after Gastro-Enterostomy.**
**Dec. 16,** Dr. Beddard, **Medicine, VI.**
**Dec. 17,** Mr. Keetley, **Clinical Lecture, II.**
**Dec. 18,** Mr. Pardoe, **The Prophylaxis of Venereal Diseases.**

**LONDON SCHOOL OF CLINICAL MEDICINE, Seamen's Hospital, Greenwich, S.E.**
**At 4 p.m.**
**Dec. 14,** Mr. Lawrence, **Hearing.**

**HOSPITAL FOR CONSUMPTION AND DISEASES OF THE CHEST, Brompton, S.W.**
**At 4 p.m.**
**Dec. 16,** Dr. R. A. Young, **The Cardio-Vascular System in Cases of Pulmonary Tuberculosis.**

**MEDICAL GRADUATES' COLLEGE AND POLYCLINIC, 22 Chenies Street, W.C.**
**At 5.15 p.m.**
**Dec. 14,** Mr. Muirhead Little, **Pott's Disease.**
**Dec. 15,** Mr. J. E. Lane, **The Ætiology and Treatment of Chronic Urethral Discharges.**
**Dec. 16,** Mr. W. Stuart-Low, **Nasal Obstruction: Its Causes, Consequences, and Care.**
**Dec. 17,** Mr. Robert Jones, **The Treatment of Congenital Club Foot.**

**MOUNT VERNON HOSPITAL, FOR CONSUMPTION, Fitzroy Square, W.**
**At 5 p.m.**
**Dec. 17,** Dr. F. P. Weber, **Relations of Tuberculosis to other Diseases.**

ANSWERS TO CORRESPONDENTS.

M. C.—The Adams car mentioned by our correspondent is certainly very suitable for town work, mainly for the reasons he gives: in fact we understand that it is possible to run this car backwards and forwards without any perceptible pause between the two movements.

"H. P." writes: I would be grateful if you or any of your readers could give me the title and net price of any work containing an account of Professor Schäfer's system of artificial respiration.

THE proprietors of "Oxo" and "Lemco" inform us that they propose to send a few gentlemen, free of expense, on a voyage of 14,000 miles to the Oxo Cattle Farms and back to enable them to see for themselves the gigantic organisation which the Oxo Company have built up during the past forty-three years. The voyage will be undertaken in the first-class saloon of one of the finest steamships in the world, and the fortunate travellers will be afforded a fortnight's tour by rail and coach on the Oxo Cattle Farms. The Oxo Company publish an illustrated pamphlet, giving full particulars, which will be sent free to any of our readers who are interested, on application to "Oxo," 4 Lloyd's Avenue, London, E.C.

THE HOSPITAL DECEMBER 12, 1908.

*Name* ......................................................

*Address* ......................................................

......................................................

This Coupon must accompany manuscript or contributions intended for THE HOSPITAL.

